# Glucocorticoid-Induced Leucine Zipper Alleviates Lung Inflammation and Enhances Bacterial Clearance during Pneumococcal Pneumonia

**DOI:** 10.3390/cells11030532

**Published:** 2022-02-03

**Authors:** Jéssica Amanda Marques Souza, Antônio Felipe S. Carvalho, Lais C. Grossi, Isabella Zaidan, Leonardo Camilo de Oliveira, Juliana P. Vago, Camila Cardoso, Marina G. Machado, Geovanna V. Santos Souza, Celso Martins Queiroz-Junior, Eric F. Morand, Stefano Bruscoli, Carlo Riccardi, Mauro M. Teixeira, Luciana P. Tavares, Lirlândia P. Sousa

**Affiliations:** 1 Signaling in Inflammation Laboratory, Departamento de Análises Clínicas e Toxicológicas, Faculdade de Farmácia, Universidade Federal de Minas Gerais, Belo Horizonte 31270-901, Brazil; jessicaamanda.biomed@gmail.com (J.A.M.S.); afs.carvalho@hotmail.com (A.F.S.C.); laiscgf@gmail.com (L.C.G.); bellazaidanmoreira@gmail.com (I.Z.); camila.faceg@gmail.com (C.C.); marinagm.biomed@gmail.com (M.G.M.); geovanna_valadares@hotmail.com (G.V.S.S.); 2 Programa de Pós-Graduação em Ciências Farmacêuticas, Faculdade de Farmácia, Universidade Federal de Minas Gerais, Belo Horizonte 31270-901, Brazil; 3 Centro de Pesquisa e Desenvolvimento de Fármacos, Departamento de Bioquímica e Imunologia, Instituto de Ciências Biológicas, Universidade Federal de Minas Gerais, Belo Horizonte 31270-901, Brazil; leonardocamilo.oliveira@yahoo.com.br (L.C.d.O.); cmqj@yahoo.com.br (C.M.Q.-J.); mmtex.ufmg@gmail.com (M.M.T.); 4 Experimental Rheumatology, Department of Rheumatology, Radboud Institute for Molecular Life Sciences, Radboud University Medical Center, 6525 GA Nijmegen, The Netherlands; julypri@gmail.com; 5 Rheumatology Group, Centre for Inflammatory Diseases, School of Clinical Sciences at Monash Health, Monash University, Melbourne 3168, Australia; eric.morand@monash.edu; 6 Department of Medicine and Surgery, Section of Pharmacology, University of Perugia, 06156 Perugia, Italy; stefano.bruscoli@unipg.it (S.B.); carlo.riccardi@unipg.it (C.R.); 7 Pulmonary and Critical Care Medicine Division, Department of Medicine, Brigham and Women’s Hospital and Harvard Medical School, Boston, MA 02115, USA; lupt26@gmail.com

**Keywords:** inflammation resolution, proresolving mediators, *Streptococcus pneumoniae*, acute lung injury

## Abstract

Pneumonia is a leading cause of morbidity and mortality. While inflammation is a host protective response that ensures bacterial clearance, a finely regulated response is necessary to prevent bystander tissue damage. Glucocorticoid (GC)-induced leucine zipper (GILZ) is a GC-induced protein with anti-inflammatory and proresolving bioactions, yet the therapeutical role of GILZ in infectious diseases remains unexplored. Herein, we investigate the role and effects of GILZ during acute lung injury (ALI) induced by LPS and *Streptococcus pneumoniae* infection. GILZ deficient mice (GILZ^−/−^) presented more severe ALI, characterized by increased inflammation, decreased macrophage efferocytosis and pronounced lung damage. In contrast, pulmonary inflammation, and damage were attenuated in WT mice treated with TAT-GILZ fusion protein. During pneumococcal pneumonia, TAT-GILZ reduced neutrophilic inflammation and prevented the associated lung damage. There was also enhanced macrophage efferocytosis and bacterial clearance in TAT-GILZ-treated mice. Mechanistically, TAT-GILZ enhanced macrophage phagocytosis of pneumococcus, which was lower in GILZ^−/−^ macrophages. Noteworthy, early treatment with TAT-GILZ rescued 30% of *S. pneumoniae*-infected mice from lethal pneumonia. Altogether, we present evidence that TAT-GILZ enhances host resilience and resistance to pneumococcal pneumonia by controlling pulmonary inflammation and bacterial loads leading to decreased lethality. Exploiting GILZ pathways holds promise for the treatment of severe respiratory infections.

## 1. Introduction

Pneumonia, mainly caused by *Streptococcus pneumoniae*, is the leading cause of death among children in the world, being responsible for up to 14% of deaths in children under 5 years of age [1]. *S. pneumoniae* is an extracellular Gram-positive pathobiont that colonizes mucous surfaces of the superior respiratory tract. Under certain circumstances, *S. pneumoniae* can disseminate to the lungs and blood causing severe invasive disease [2,3]. Severe pneumococcal pneumonia is characterized by an intense inflammatory response with massive recruitment and activation of leukocytes leading to significant pulmonary damage and bacteria dissemination [4,5]. More than 100 different serotypes of *S. pneumoniae* have been identified, representing an important challenge for the development of vaccines and therapeutic strategies to control the health burden of pneumococcal infections [6]. Treatment strategies focused on the host immune responses are less susceptible to strain differences and induction of antimicrobial resistance [4].

Resolution of inflammation is an active process that occurs in a coordinated way to guarantee the return to tissue homeostasis after an injurious stimulus. In the lungs, resolution of inflammation ensures restoration of normal vascular permeability, cessation of granulocyte recruitment after pathogen clearance, enhance macrophage migration and removal of apoptotic cells and debris, and induces repair responses that lead to the return of normal function of the organ [7]. Therefore, exploiting proresolving molecular and cellular circuits are potentially beneficial for treating pulmonary infections at least as adjunctive therapy [8,9,10,11].

The glucocorticoid-induced leucine zipper (GILZ) is a protein induced by glucocorticoids and has a key role in the control of immune responses [12,13]. GILZ can interact with and inhibit pro-inflammatory transcription factors, such as NF-κB and AP-1, and other signaling molecules, including Raf-1, Ras and ERK1/2 [12,14,15]. It has been demonstrated that over-expression of GILZ or administration of TAT-GILZ (a cell permeable GILZ-fusion protein) promoted anti-inflammatory and proresolving actions, by increasing apoptosis of neutrophils and shortening resolution intervals [16], by modulating macrophage polarization and apoptotic cell clearance by efferocytosis [13] and by enhancing bacterial clearance in a model of polymicrobial sepsis [17,18]. Conversely, the immunomodulatory role of GILZ is evidenced by uncontrolled persistent inflammation triggered in GILZ-deficient mice in experimental models of inflammatory diseases [19].

Despite the growing evidence supporting the anti-inflammatory and proresolving bioactions of GILZ, its role during infection, especially in the lungs, is yet to be described. Here, we investigate the effect of TAT-GILZ treatment during acute lung injury (ALI) induced by LPS and in a relevant model of pneumococcal pneumonia. We report that TAT-GILZ modulates inflammation and promotes efferocytosis and bacterial clearance resulting in decreased lung damage and barrier breech during pneumonia, while enhancing macrophages phagocytosis of bacteria, suggesting that the exploitation of GILZ-based therapeutics holds promise in the treatment of severe pneumonia.

## 2. Materials and Methods

### 2.1. Mice

Male BALB/c mice (8–10 weeks old) were obtained from the central animal facility of Universidade Federal de Minas Gerais and maintained with free access to commercial chow and water. GILZ-deficient male mice (GILZ^−/−^) and their C57BL/6 WT littermates were bred in the animal facility of the Immunopharmacology Laboratory and generated as described [20]. The GILZ gene is X chromosome linked and male GILZ^−^^/−^ mice are infertile [21,22]. Therefore, heterozygous females are crossed with GILZ^+/+^ males. Littermates from different breeding, matched for age and weight were used. All procedures described were approved by the local animal ethics committee (CEUA 162/2020).

### 2.2. Bacterial Strain

ATCC 6303 serotype 3 was grown in Todd Hewitt Broth 0.5% of yeast extract (THY broth) 37 °C with 5% CO_2_ until log phase (optical density at 600 nm = 0.4). Bacterial inoculum was prepared in sterile PBS as previously described [8,11]. In all experiments, inoculum was confirmed by plating of bacterial suspension.

### 2.3. Acute Lung Injury (ALI) Models and Treatments Protocols

Mice were anesthetized with ketamine/xylazine (80 mg/kg and 10 mg/kg, respectively) and ALI was induced by LPS or *Streptococcus pneumoniae* instillation. 1 μg (500 endotoxin unit) of LPS or 10^5^ CFU of *S. pneumoniae* were instilled intranasally in 40 µL. Control mice received saline (mock infection). The model of ALI induced by LPS was previously standardized in our laboratory and the peak of inflammation was shown to be between 12 to 24 h (Supplementary Figure S1). The model of pneumococcal pneumonia was performed using male BALB/c mice as described previously [8,11] and ALI model was performed in male C57BL/6, given that is the background of GILZ^−^^/−^ mice. Twelve hours post infection or LPS instillation, mice were treated with 200 µL of TAT-GILZ (0.2 mg/kg, i.p.) or empty TAT (0.1 mg/kg, i.p.). Proteins were dissolved in DMSO and diluted further in sterile saline. TAT and TAT-GILZ dosage were chosen according to previously published studies [13,16,19,23,24]. Equimolar doses of TAT and TAT-GILZ were injected, considering that the molecular weight (MW) of TAT is half of TAT-GILZ. Control mice received sterile saline only. The peptide TAT and the TAT-GILZ fusion protein, constructed by inserting GILZ cDNA in the TAT-C vector to produce an in-frame fusion protein, were generated as previously described [16,19,23]. Each batch of TAT and TAT-GILZ, was prepared in LPS free conditions [13,23]. Twenty-four hours post-LPS instillation or pneumococcus infection, lungs and blood (from the abdominal vena cava) were harvest for further analysis. To analyze survival rates, mice were treated at 12, 24 and 48 h after *S. pneumoniae* infection.

### 2.4. Bronchoalveolar Lavage (BAL) and Tissue Extraction

At euthanasia timepoints, blood was collected for bacteria counts (in pneumococcal model) and cytokine/chemokine evaluation. Next, BAL was performed in both lungs, simultaneously, by exposing the trachea and inserting a 1.7 mm catheter. Two aliquots of 1 mL of PBS were flushed three times into the bronchoalveolar compartment to recover the leukocytes and bacteria in the airways. Subsequently, the left lung was reserved in neutral buffered formalin (10%) for histological analysis. Then, 50 µL of BAL was submitted to serial dilution and plated in blood agar for bacterial counts. After centrifugation, the pellet of cells was used for total and differential cell counts. Cytocentrifuge preparations (Shandon III) stained with May-Grunwald-Giemsa were used for differential counts of leukocytes, based on morphological criteria. BAL fluid supernatants were used for cytokine evaluation by ELISA and total protein quantification using the Bradford assay (Bio-Rad Laboratories, Hercules, CA, USA).

### 2.5. ELISA

Concentrations of TNF-α, IL-6, CXCL-1 and CXCL-2 were measured in the supernatants obtained from the bronchoalveolar lavages (BAL) according to the procedures supplied by the manufacturer (R&D Systems, Minneapolis, MN, USA).

### 2.6. Histological Analysis

Left lungs were fixed in neutral buffered formalin (10%) for 48 h and then gradually dehydrated in ethanol and embedded in paraffin. 5μm sections were cut and stained with H&E for examination under light microscopy. Histopathological score was performed as described [25] by a pathologist blinded to the experimental groups, and evaluated the following categories: airways inflammation (0–4), vascular inflammation (0–4), parenchymal inflammation (0–5) and PMN infiltrate (0–5), making a total score of 18 points.

### 2.7. Bone Marrow-Derived Macrophages (BMDMs)

Wild type C57BL/6 mice were euthanized, and tibias and femurs were collected for isolation of bone marrow. The cell suspension obtained was then centrifuged for 5 min at 1.200× *g*. The pellet was resuspended in complete conditioned media for BMDM differentiation (RPMI with 10% heat-inactivated fetal bovine serum plus and 30% L929 cell-conditioned medium), seeded on flasks and incubated at 37 °C and 5% CO_2_. After 7 days, the supernatant was removed, and adherent macrophages were detached by using a cell scraper and plated (2 × 10^5^ cells/well) in 96-well plates for phagocytosis assay.

### 2.8. Phagocytosis Assays

Phagocytosis was evaluated as previously described [11,26]. Briefly, 2 × 10^5^ alveolar macrophages AMJ2-C11 (ATCC CRL-2456) or BMDMs isolated from WT and GILZ^−^/^−^ mice were plated, and incubated with *S. pneumoniae* (MOI 1:10) for 3 h to allow phagocytosis (1 h of adhesion at 4 °C followed by 2 h at 37 °C and 5% CO_2_). Noninternalized bacteria were washed out with penicillin and streptomycin (30 µg/mL in PBS by 30 min). To assess phagocytosed bacteria, macrophages were lysed as described [11,26] and viable internalized bacteria were counted in blood agar plates after overnight incubation at 37 °C and 5% CO_2_. Pre-treatment with TAT or TAT-GILZ were by 18 h before the experiments and the dose was based in previous published studies [13,27].

### 2.9. Assessment of Efferocytosis

BAL leukocytes (5 × 10^4^) were cyto-centrifuged onto slides and stained with May-Grünwald-Giemsa. The identification and counts of macrophages with engulfed apoptotic bodies was performed by light microscopy (100× objective -(500 cells/slides). Results are expressed as the mean ± SEM of the percentage of macrophages with apoptotic cells inside [13,26].

### 2.10. Statistical Analysis

**Figure 1:**
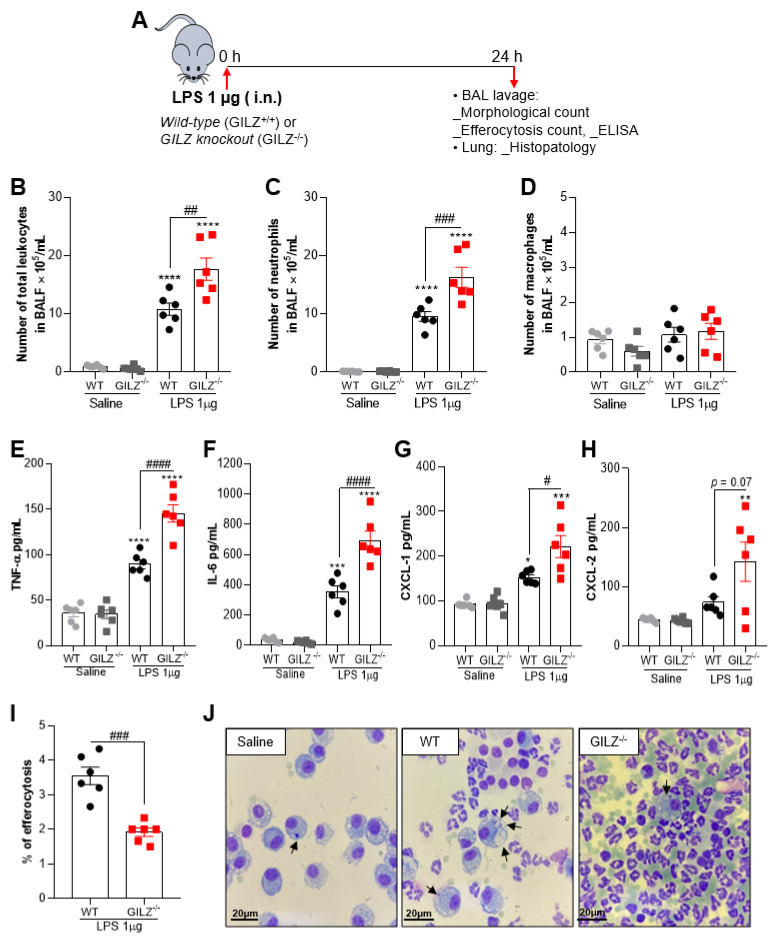
GILZ deficiency causes a greater inflammatory response to LPS-induced Acute Lung Injury. C57BL/6 WT or GILZ^−/−^ mice were stimulated with LPS (1 µg, i.n.) and euthanized 24 h later (schematic strategy in (**A**)). BAL was harvested to quantify the number of total leukocytes (**B**), neutrophils (**C**) and macrophages (**D**). Levels of the cytokines TNF-α (**E**) and IL-6 (**F**) and chemokines CXCL-1 (**G**) and CXCL-2 (**H**) were measured by ELISA in BAL fluid. Graph (**I**) shows the percentage of efferocytosis by morphological counting of cytospin slides stained with May-Grunwald-Giemsa. In (**J**), arrows indicate apoptotic neutrophils inside macrophages. Magnification 100×. Data are mean ± SEM of N = 6 animals per group. * *p* < 0.05, ** *p* < 0.01, *** *p* < 0.001 or **** *p* < 0.0001 when compared to the saline group; or as indicated: *^#^ p* < 0.05, ^##^ *p* < 0.01 *^###^ p* < 0.001 or ^####^ *p* < 0.0001 when comparing LPS-challenged GILZ^−/−^ to WT mice, by 2-way ANOVA.

**Figure 2:**
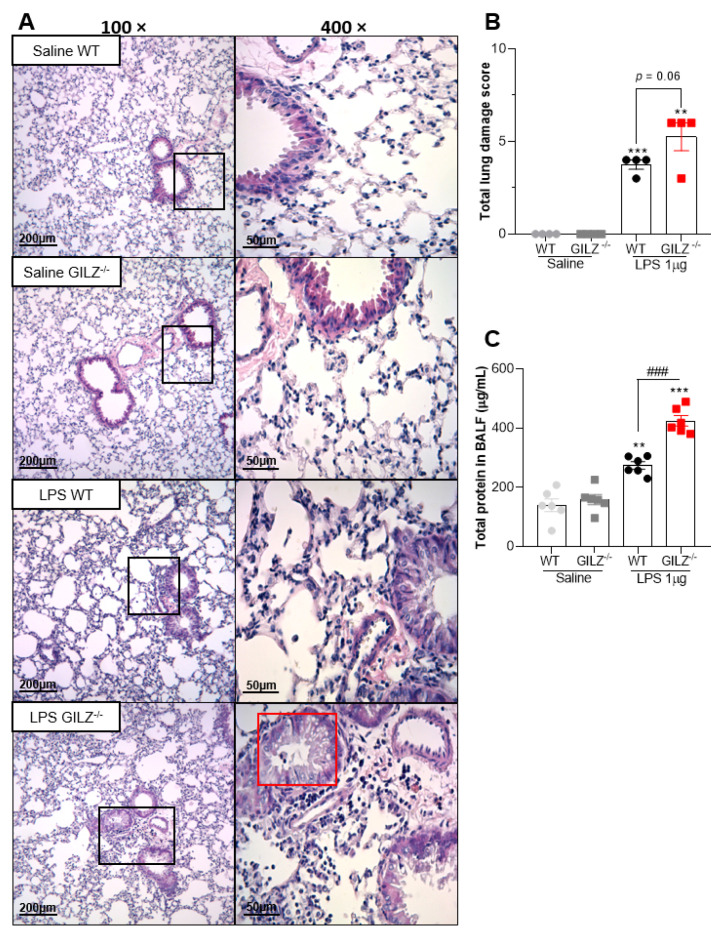
GILZ deficient mice show exacerbation of LPS-induced Acute Lung Injury. C57BL/6 WT or GILZ^−/−^ mice were stimulated with LPS (1 µg, i.n.) and euthanized 24 h later. Representative slides of hematoxylin and eosin (H&E) stained lungs are shown (**A**). Scale bars = 200 µm (low magnification) and 50 µm (high magnification). Right slides are higher magnifications (400×) of the selected areas (boxes) in left slides (100×). Histopathological score evaluated airway, vascular, and parenchymal inflammation, neutrophilic infiltration, and epithelial injury (**B**). A red box represents bronchiolar epithelium degeneration, seen only in LPS-instilled GILZ^−^^/−^ group of mice. The levels of total protein in BALF were evaluated (**C**). Data are mean ± SEM of N = 4 animals (histopathology) or N = 6 (protein levels) per group. ** *p* < 0.01 or *** *p* < 0.001 when compared to saline instilled groups; or as indicated: ^###^ *p* < 0.001 by 2-way ANOVA or *p* = 0.06 when comparing LPS-challenged GILZ^−^^/−^ to WT mice (*t* test).

Statistics were performed using GraphPad Prism 8.0. Two-way (Figure 1 and Figure 2) followed by Tukey correction or one-way ANOVA, followed by Newman-Keuls post-test, were used to compare more than two groups. Unpaired *t*-test was used for comparisons between two groups. The survival curves were analyzed by Log-rank test. Results with *p* < 0.05 were considered statistically significant.

## 3. Results

### 3.1. GILZ Is Crucial for Inflammation Control during LPS-Induced ALI

To uncover the role of GILZ in pulmonary inflammation and injury, we have established a murine mild model of ALI that exhibits intense neutrophil infiltration within 12–24 h of the intranasal challenge with LPS but resolves thereafter (Supplementary Figure S1A–C) and investigated the inflammatory response in GILZ deficient mice (GILZ^−/−^) compared to WT littermates (schematic experimental design in A). Interestingly, GILZ^−/−^ mice presented increased numbers of leukocytes (Figure 1B), especially neutrophils (Figure 1C), when compared to their wild-type (WT) littermates. Macrophage numbers were not modified by LPS challenge (Figure 1D). The concentrations of TNF-α and IL-6 were increased in BAL fluid after LPS instillation and was markedly higher in GILZ^−/−^ mice (Figure 1E,F). Alongside increased numbers of neutrophils found in GILZ^−/−^ mice, the levels of the neutrophil active chemokines CXCL-1 and CXCL-2 were also higher in GILZ^−/−^ mice (Figure 1G,H). Noteworthy, there was decreased macrophage efferocytosis, as seen by counting the percentage of macrophage that had ingested apoptotic neutrophils on cytospin slides, in GILZ^−/−^ mice (Figure 1I and representative images in Figure 1J), consistent with defective clearance of apoptotic neutrophils in the latter animals.

Histopathological analysis of the lungs 24 h after LPS showed that GILZ^−/−^ challenged mice had more severe lung damage and increased infiltration of inflammatory cells as compared to WT mice (Figure 2A). In addition, deficiency of GILZ led to marked bronchiolar epithelium degeneration at 24 h after LPS instillation (red box in Figure 2A), which was observed only in LPS-instilled GILZ^−/−^ mice. Group analysis showed that GILZ^−/−^ mice had more severe lung damage after LPS than the WT group (Figure 2B). No histological signs of damage were observed in lungs of saline-instilled mice (Figure 2B). Akin to the increased lung injury, the levels of total protein in BALF, an indirect measurement of edema, were higher in GILZ^−/−^ mice than in their WT littermates (Figure 2C). Taken together, these data show that in the absence of GILZ, LPS instillation causes greater overall lung damage and disruption of the epithelial barrier.

**Figure 3:**
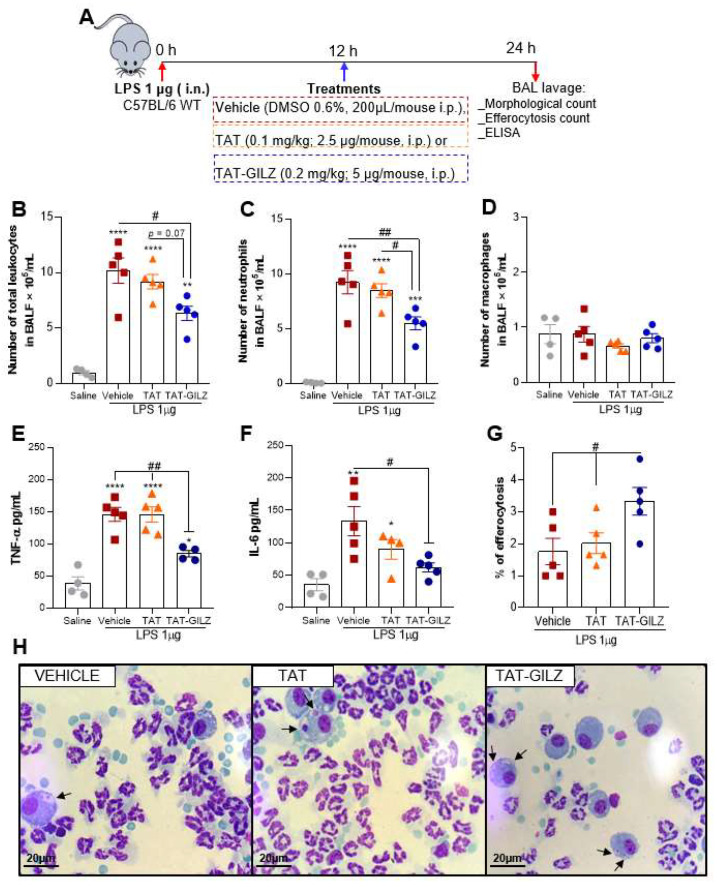
TAT-GILZ treatment during LPS-induced ALI modulates neutrophilic inflammation and increases efferocytosis indexes. C57BL/6 WT mice were stimulated with LPS (1 µg, i.n.), treated with TAT (0.1 mg/kg, i.p.) or TAT-GILZ (0.2 mg/kg, i.p.) 12 h p.i., and euthanized 24 h later (schematic protocol in (**A**)). BAL was harvested to quantify number of total leukocytes (**B**), neutrophils (**C**) and macrophages (**D**). Levels of the cytokines TNF-α (**E**) and IL-6 (**F**) were measured by ELISA in BAL fluid. Graph (**G**) shows the percentage of efferocytosis by morphological counting of cytospin slides stained with May-Grunwald-Giemsa. In (**H**), arrows indicate apoptotic neutrophils inside macrophages. Magnification 100×. Vehicle group received DMSO 0.6%. Data are mean ± SEM of N = 4–5 animals per group. * *p* < 0.05, ** *p* < 0.01, *** *p* < 0.001 or **** *p* < 0.0001 when compared to saline instilled groups; or as indicated: ^#^ *p* < 0.05, ^##^ *p* < 0.01 or *p* = 0.07 when comparing TAT-GILZ-treated LPS-challenged mice to vehicle or TAT groups, by 1-way ANOVA.

Next, we wondered whether exogenous administration of GILZ would protect mice from LPS-induced inflammation and damage. LPS-instilled mice were treated with TAT-GILZ, a cell-permeable GILZ fusion protein, at the peak of inflammation (schematic therapeutic protocol show in Figure 3A). We observed that TAT-GILZ treatment was associated with decreased leukocyte counts in BAL (Figure 3B), especially neutrophils (Figure 3C), without changing macrophage numbers (Figure 3D). In line with the reduction of neutrophilic inflammation, TAT-GILZ also decreased TNF-α and IL-6 levels in BAL fluid of mice instilled with LPS (Figure 3E,F). Notably, treatment with TAT-GILZ also increased the percentage of efferocytosis (graphed in Figure 3G and representative images in Figure 3H). In summary, LPS-induced ALI was alleviated by TAT-GILZ treatment through the modulation of inflammation and induction of macrophage efferocytosis, a key determinant for the resolution of neutrophilic inflammation [28,29].

### 3.2. TAT-GILZ Treatment Modulates the Inflammatory Response, Enhances Cell Efferocytosis and Bacterial Clearance in Pneumococcal Pneumonia

**Figure 4:**
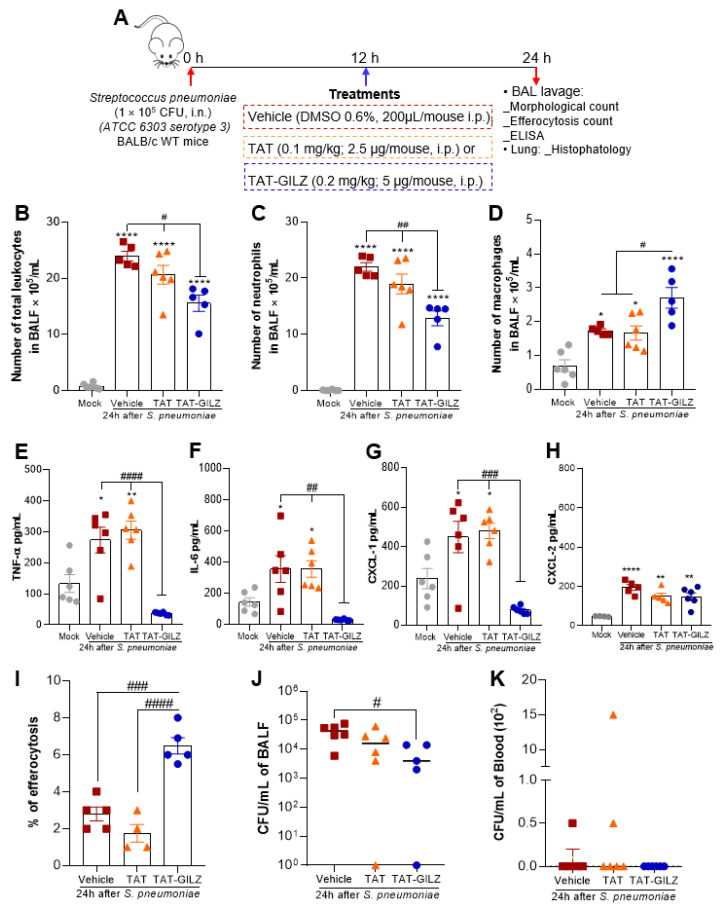
Effect of treatment with TAT-GILZ in the course of pneumococcal pneumonia. BALB/c mice were infected with *S. pneumoniae* (1 × 10^5^ CFU, i.n.) and then treated with TAT (0.1 mg/kg, i.p.) or TAT-GILZ (0.2 mg/kg, i.p.) 12 h p.i. Mice were euthanized 24 h p.i. (schematic protocol in (**A**)). Quantification of the number of total leukocytes (**B**), neutrophils (**C**), and macrophages (**D**) in BAL. Measurements of cytokines TNF-α (**E**), IL-6 (**F**) and the chemokines CXCL-1 (**G**) and CXCL-2 (**H**) were performed by ELISA in BAL fluid. Graph (**I**) shows the percentage of efferocytosis (macrophage that had ingested apoptotic cells). Bacterial counts in BALF (**J**) and blood (**K**) were also evaluated. The Mock group (uninfected) received salina and vehicle group received DMSO 0.6%. Data are mean ± SEM of N = 5–6 animals per group. * *p* < 0.05, ** *p* < 0.01 or **** *p* < 0.0001 when compared to the mock group (saline instilled); or as indicated by ^#^ *p* < 0.05, ^##^ *p* < 0.01, ^###^ *p* < 0.001 or ^####^ *p* < 0.0001 when comparing TAT-GILZ-treated pneumococcal pneumonia to vehicle or TAT groups, by 1-way ANOVA.

Given the role of GILZ in modulating pulmonary inflammation in LPS-induced ALI and that exacerbated inflammation is correlated with disease severity in pneumonia [30,31], we wondered whether exogenous administration of GILZ could afford protection in an model of ALI caused by *S. pneumoniae* infection. To address that, mice were intranasally infected with 10^5^ CFU of *S. pneumoniae* and treated, 12 h later, with TAT-GILZ (0.2 mg/kg, i.p.). Mice treated with TAT alone (0.1 mg/kg) or vehicle were used as infected control groups. At 24 h after infection, the animals were euthanized, and the inflammatory parameters evaluated (schematic experimental design in Figure 4A). Systemic treatment with TAT-GILZ significantly decreased the overall leukocyte recruitment to the airways (Figure 4B), which was mainly composed of neutrophils (Figure 4C). Although fewer than neutrophils, macrophage numbers were increased by TAT-GILZ treatment (Figure 4D). Treatment with TAT-GILZ also reduced concentration of TNF-α and IL-6 and the neutrophil chemoattractant chemokine CXCL-1 (Figure 4E–G), having no effect on CXCL-2 levels (Figure 4H). These results suggest that the decrease in inflammatory cell infiltrate to the alveoli in response to TAT-GILZ is accompanied by a reduction in the local production of pro-inflammatory mediators.

Since we have observed anti-inflammatory effects of TAT-GILZ treatment in pneumococcal pneumonia, we next evaluated the effects of the protein on bacteria control and apoptotic cell clearance by macrophages. TAT-GILZ significantly increased efferocytosis by macrophages (Figure 4I), an important marker for proper resolution of lung inflammation [28]. In addition, TAT-GILZ, but not TAT alone, decreased BAL bacteria counts (Figure 4J). Consistently with previous reports [11], at 24 h post-infection, bacteria are rarely found in the blood of mice (Figure 4K). Collectively, these data indicate that treatment with TAT-GILZ modulated inflammatory infiltration to the lungs during infection, but despite the reduction in inflammation, TAT-GILZ treatment reduced BAL bacteria counts.

### 3.3. Treatment with TAT-GILZ Attenuates Lung Damage Caused by Pneumococcal Infection

**Figure 5:**
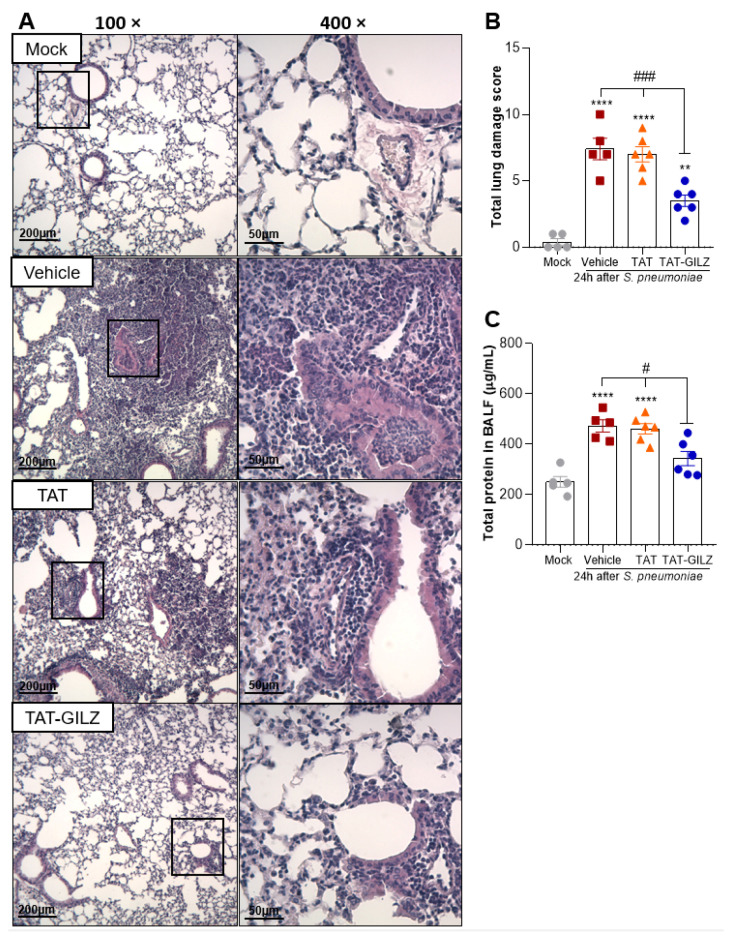
Effect of treatment with TAT-GILZ in lung damage caused by pneumococcal infection. BALB/c mice were infected with *S. pneumoniae* (1 × 10^5^ CFU, i.n.), and then treated with TAT (0.1 mg/kg, i.p.) or TAT-GILZ (0.2 mg/kg, i.p.) 12 h p.i., and euthanized 24 h p.i. Mock group (uninfected) received salina and vehicle group received DMSO 0.6%. Representative slides of hematoxylin and eosin (H&E) stained lungs are shown (**A**). Scale bars = 200 µm (low magnification) and 50 µm (high magnification). Right slides are higher magnifications (400×) of the selected areas (boxes) in left slides (100×). Histopathological score evaluated airway, vascular, and parenchymal inflammation, neutrophilic infiltration, and epithelial injury (**B**). The total protein level in BAL is show in (**C**). Data are mean ± SEM of N = 5–6 animals per group. ** *p* < 0.01, **** *p* < 0.0001 when compared to mock group, or as indicated: ^#^ *p* < 0.05, ^###^ *p* < 0.001 when comparing TAT-GILZ-treated pneumococcal pneumonia mice to vehicle or TAT groups, by 1-way ANOVA.

Histological analysis of lungs 24 h post-*S. pneumoniae* infection showed considerable pneumonia characterized by marked lung damage and striking infiltration of inflammatory cells in bronchioles and alveoli of mice (Figure 5A). Keeping with the overall reduction in inflammation and bacteria counts in BAL, TAT-GILZ-treated mice presented mild pneumonia with preservation of lung architecture and reduction of leukocyte infiltration and pulmonary consolidation (Figure 5A). Histopathological score showed that while a higher proportion of vehicle and TAT-treated mice presented severe to moderate pneumonia, all the mice in TAT-GILZ showed mild histology signs of pneumonia (Figure 5B).

Keeping with the reduction in lung injury, the levels of total protein in BALF, an indirect measurement of edema, were also lower in animals treated with TAT-GILZ, compared to other infected groups (Figure 5C). Taken together, these data suggest that regulation of both inflammation and bacterial loads by TAT-GILZ reduced overall lung injury.

### 3.4. GILZ Stimulates Alveolar and Bone Marrow-Derived Macrophages to Phagocytose Pneumococcus

**Figure 6:**
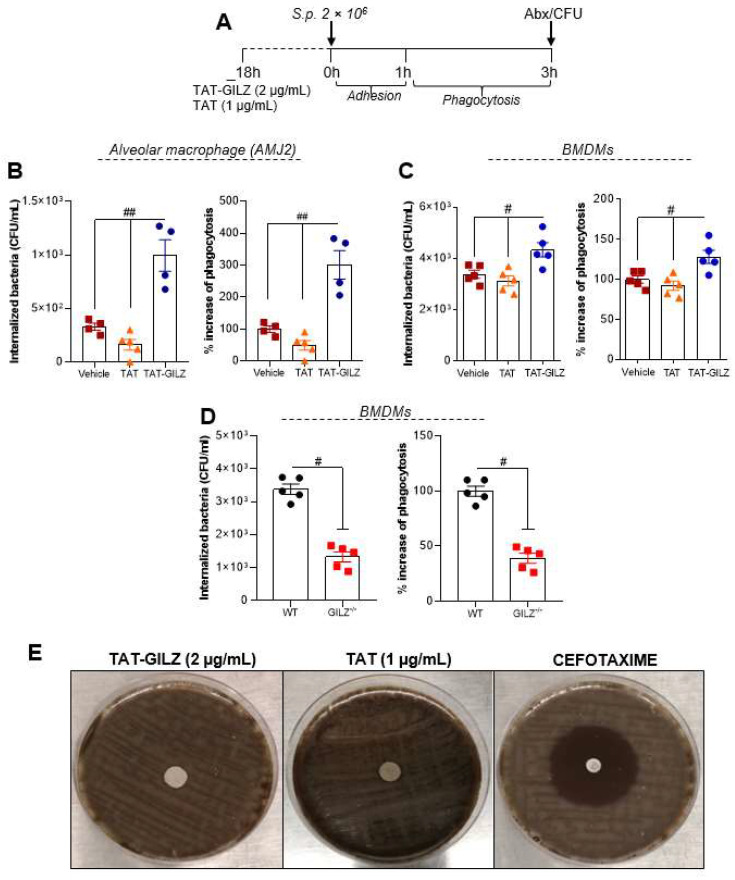
TAT-GILZ enhanced macrophages phagocytosis of *Streptococcus pneumoniae.* Phagocytosis of bacteria–Experimental design in (**A**)–were evaluated in alveolar macrophages (AMJ2-C11) (**B**) or BMDMs obtained from WT mice (**C**). Macrophages (2 × 10^5^) were pre-treated with TAT (1 μg/mL) or TAT-GILZ (2 μg/mL) for 18 h and then incubated with *S. pneumoniae* (MOI 1:10) for 3 h to allow adhesion and phagocytosis. Noninternalized bacteria were excluded by the incubation of the cells with penicillin/streptomycin (Abx), followed by the lysis of macrophages to identify the number of viable phagocytosed bacteria. In other experimental group BMDMs from naïve WT and GILZ^−/−^ (2 × 10^5^) were also subjected to phagocytosis as describe bellow (**D**). Results are expressed as CFU of internalized bacteria or % of phagocytosis (CFU counts on blood agar plates, N = 4–5) and are presented as mean ± SEM; ^#^ *p* < 0.05, ^##^ *p* < 0.01 when comparing TAT-GILZ-treated cells to vehicle or TAT, by 1-way ANOVA (B–C). Comparison between BMDMs from WT and GILZ^−/−^ were by *t*-test (^#^ *p* < 0.05). Data are representative of 3 independent experiments performed in biological quadruplicates or quintuplicates. TAT-GILZ (2 μg/mL), TAT (1 μg/mL) or cefotaxime (30 μg—control) impregnated on sterile filters were then added to blood agar plates containing *S. pneumoniae*. The Clinical and Laboratory Standards Institute (CLSI) only defines MIC values for cephalosporins. However, here we have performed the disc diffusion method using a cefotaxime disc for comparative purposes. The zone of growth inhibition was evaluated after 18 h incubation at 37 °C and 5% CO_2_ (**E**).

Since TAT-GILZ treated mice presented lower bacteria counts in BAL and increased macrophage numbers, we next evaluated whether TAT-GILZ could directly enhance macrophage phagocytosis of bacteria. To assess this, alveolar macrophages (AMJ2-C11) were pre-treated with TAT (1 µg/mL) or TAT-GILZ (2 µg/mL) for 18 h, followed by infection with 2 × 10^6^ CFU of *S. pneumoniae* for 3 h (Experimental design in Figure 6A), and phagocytosed bacteria by alveolar macrophages were enumerated. TAT-GILZ treatment was associated with increased bacterial phagocytosis compared to cells treated with vehicle or TAT (Figure 6B). The same strategy was carried out in bone-marrow derived macrophages (BMDMs) with similar findings (Figure 6C).

We next evaluated whether endogenous GILZ impacted on the phagocytic capacity of macrophages. BMDMs from GILZ^−/−^ mice or WT littermates were infected with 2 × 10^6^ CFU of *S. pneumoniae* for 3 h, and phagocytosis was evaluated as above (Figure 6A and methods). Interestingly, cells from GILZ^−/−^ animals showed significant lower numbers of internalized bacteria than BMDMs from WT mice (Figure 6D). Direct anti-bacterial actions of GILZ were investigated next by plating *S. pneumoniae* on blood agar plates and adding TAT-GILZ, TAT, or the antibiotic cefotaxime as control. TAT-GILZ had no direct bactericidal effect (Figure 6E). Together, these data suggest that GILZ stimulates host cellular anti-bacterial mechanisms and that treatment with TAT-GILZ is a potential therapeutic strategy for enhancing host resistance to infection.

### 3.5. TAT-GILZ Treatment Rescue Mice from Lethality Caused by Pneumococcal Pneumonia

**Figure 7:**
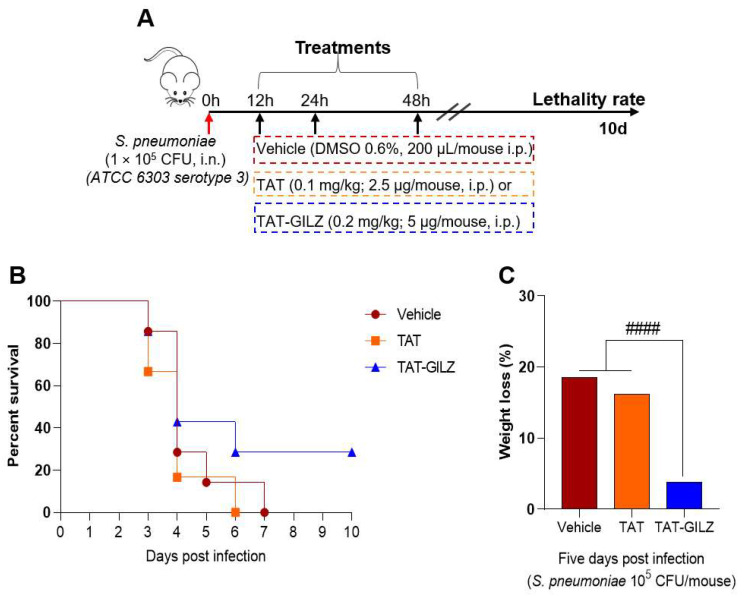
TAT-GILZ treatment rescued 30% of mice from lethal pneumonia. BALB/c mice were infected with *S. pneumoniae* (1 × 10^5^ CFU, i.n.), treated with TAT (0.1 mg/kg, i.p.) or TAT-GILZ (0.2 mg/kg, i.p.) at 12, 24 and 48 h p.i. (**A**), the groups were monitored for lethality rates (**B**) percentage of weight lost at day five (**C**). Vehicle group received DMSO 0.6%. N = 6–7 animals per group. ^####^ *p* < 0.0001 when comparing TAT-GILZ-treated pneumococcal pneumonia mice to vehicle or TAT groups, by 1-way ANOVA.

Considering that TAT-GILZ can improve both inflammation and bacteria counts in *S. pneumoniae* infection, we investigated whether treatment could influence animal survival. For this purpose, mice were intranasally infected with 10^5^ CFU of *S. pneumoniae* and received TAT-GILZ, TAT or vehicle intraperitoneally at 12, 24 and 48 h after infection and followed for 10 days to assess survival and weight. All control treated mice died by day 6, but treatment with TAT-GILZ resulted in 30% of mice surviving (Figure 7A). Additionally, while vehicle and TAT-treated animals showed high percentage of weight lost at day five, the group of mice treated with TAT-GILZ had a minor weight loss (Figure 7C) and started to regain weight on the sixth day, presenting higher mobility, signs of faster recovery.

## 4. Discussion

Pneumonia is defined as an inflammation of the lung parenchyma and airways triggered by pathogens such as bacteria, viruses, fungi, or others. Despite the availability of antibiotics and vaccines, pneumococcal pneumonia is a leading cause of mortality, especially in children and the elderly [30,31]. Inflammation protects the host by eliminating the infectious agent, but must be self-limited, progressing to complete resolution [7,15,32]. Inflammation resolution is an active process that involves the production and activation of biochemical mediators and signaling pathways to ensure the restoration of tissue homeostasis [32,33,34]. On the other hand, exacerbated inflammatory responses triggered by infection can cause intense pulmonary damage and dysfunction increasing pneumonia severity [35].

Glucocorticoids (GCs) are important drugs for the treatment of many inflammatory diseases and have been proven beneficial to treat inflammation caused by certain infectious diseases, including meningitis, tuberculosis and bacteria pneumonia [36], and more recently COVID-19 [37]. Indeed, preclinical studies have demonstrated that the combination of GCs with antibiotics prevents bystander inflammation-related lung damage during pneumococcal pneumonia [38,39]. However, long term or high dose GC treatment is generally associated to important side effects.

**Figure 8:**
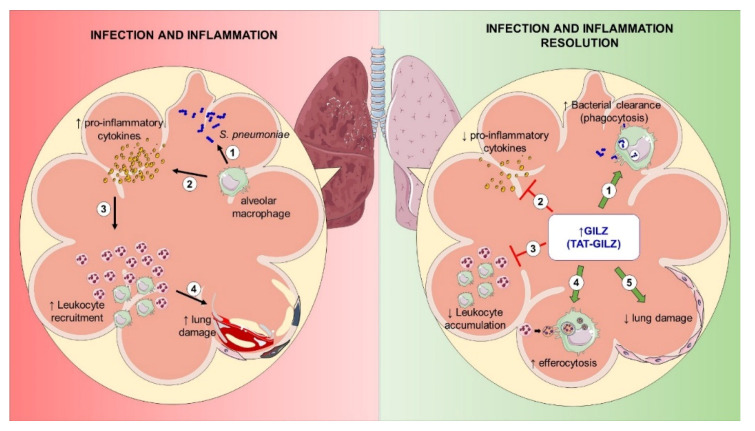
Proresolving effects of the TAT-GILZ treatment in pulmonary infection and inflammation. During severe pneumococcal infection (left panel), alveolar macrophages (1) recognize and sign bacteria through substantial release of pro-inflammatory mediators (2) that activate the endothelium and increase the recruitment of leukocytes (3) to the lungs. The persistent and excessive inflammation leads to significant lung damage (4). TAT-GILZ treatment (right panel), controls excessive inflammation by increasing bacterial clearance (1) (through phagocytosis of pneumococcus), decreasing the release of pro-inflammatory mediators (2) and reducing the accumulation of leukocytes (3). TAT-GILZ also increased efferocytosis of apoptotic neutrophils and (4) protected the pulmonary epithelium from the inflammatory bystander damage (5). Overall TAT-GILZ enhanced resolution of infection and inflammation during pneumococcal pneumonia.

GC-induced proteins that mediate potent immunomodulatory actions without deleterious GC effects, such as GILZ [40,41], might represent candidates for treating inflammatory diseases. In fact, the benefits of AnxA1, another proresolving GC-induced protein, in pneumonia were recently described [11] and support this concept. Treatment with the AnxA1 mimetic peptide, Ac2-26, decreased inflammation, lung damage and bacterial load in the airways, increasing bacterial phagocytosis by macrophages [11]. Few studies have so far investigated the effects of GILZ in preclinical models of infection [14,17,18]. Here, we have identified the protective role of GILZ in two models of ALI caused by LPS and *S. pneumoniae*. We show that TAT-GILZ was able to (i) decrease neutrophilic infiltration, enhance efferocytosis of apoptotic cells and bacterial clearance in the airways; (ii) attenuate lung damage; (iii) increase bacterial phagocytosis in vitro; and (iv) reduce lethality of pneumonia (Figure 8).

ALI manifests as an inflammatory process clinically characterized by pulmonary infiltrates, hypoxemia, and edema, with pneumonia being the main cause of ALI morbidity and mortality [42]. The accumulation of pro-inflammatory factors and neutrophils is an important feature of ALI. Here, even using a mild model of ALI (by instillation of 1 μg LPS) we have observed an exacerbation of LPS-induced pneumonia in the absence of GILZ, characterized by increased neutrophilic infiltration, pro-inflammatory cytokines and neutrophil active chemokines in the airways, and pronounced pulmonary damage. In addition, and in keeping with previous findings in vitro (by using BMDMs) and in vivo in a self-resolving model of pleurisy [13], GILZ is an important determinant of efferocytosis, with GILZ^−/−^ mice having reduced efferocytosis, an important hallmark of resolution of inflammation in the lung [28]. Consistent with our previous findings obtained in LPS-induced pleurisy, the treatment with TAT-GILZ decreased pulmonary neutrophilic inflammation and pro-inflammatory cytokines/chemokines and increased efferocytosis during LPS-induced ALI and pneumococcal pneumonia. This is in keeping with the findings that GILZ was shown to reduce inflammation and improve survival of mice during LPS-induced endotoxemia [43] and it seems to be involved on attenuation of the systemic LPS-response induced by short-chain alcohols [44]. These findings suggest that endogenous GILZ tempers inflammatory response and its exogenous administration (e.g., by TAT-GILZ) favors resolution of inflammation during LPS-induced ALI.

Previously, we have shown that expression of GILZ is increased during the resolving phase of inflammation and that administration of TAT-GILZ at the peak of inflammation promotes a decrease in the number of viable neutrophils and increased apoptotic neutrophils [16]. In addition to inducing neutrophil apoptosis, TAT-GILZ also promoted macrophage polarization and increased efferocytosis [13]. The efferocytosis of apoptotic neutrophils by macrophages is a process mediated mainly by M2-like macrophages and is a critical stage of inflammation resolution [26]. M2-like macrophages produces anti-inflammatory mediators such as IL-10 and TGF-β [26,45] and biologically active amounts of pro-resolving mediators [46]. Indeed, under M1 (IFN-γ+ LPS) polarization stimuli, BMDMs from GILZ^−/−^ mice show increased secretion of pro-inflammatory cytokines (IL-6 and TNF-α), while secretion of the anti-inflammatory cytokine IL-10 was reduced, as compared to BMDMs from WT littermates. Moreover, GILZ^−/−^ mice have more M1 macrophage numbers after LPS-induced pleurisy associated with lower rates of efferocytosis, as compared to WT mice [13]. Conversely, in this same inflammatory model, TAT-GILZ treatment decreased the pro-inflammatory cytokines IL-6 and TNF-α [16] and the M1 numbers, and increased efferocytosis [13] into the pleural cavity. In keeping with that, in the present study we have found, in the both models used, increased efferocytosis in BAL after TAT-GILZ treatment. However, at the time point we performed the experiments (24 h), we did not find increased IL-10 levels (data not shown), which agree with a previous study that measured IL-10 at several time points after pneumococcus infection and did not observe increase of this cytokine, as compared to the mock group [11]. Of note, the treatment of pneumococcal pneumonia with the Annexin A1 peptide Ac2-26, a pro-resolving mediator, did not increase IL-10, while reducing the inflammatory response, lung damage, bacterial loads and increasing phagocytosis [11]. Therefore, one can argue that other pro-resolving mediators released during efferocytosis might be contributing to resolution of inflammation [47].

As pneumococcal pneumonia is a leading cause of ALI, we utilized a clinically relevant model of pneumococcal pneumonia in mice to better comprehend the role of GILZ in host response to infection. As for the LPS-induced ALI model, pneumococcal lung infection led to a rapid neutrophil accumulation in lung tissue and increased production of cytokines. This is similar to the clinical features of severe pneumococcal pneumonia that can progress to acute respiratory distress syndrome (ARDS). Interestingly, GILZ expression is upregulated in neutrophils of severe ARDS patients suggesting GILZ as a potential counter-regulatory mechanism for the exacerbated pulmonary inflammation [48] and supported by persistent activation of neutrophils in the absence of GILZ in a model of *Candida albicans* infection [14]. Here, we found that treatment with TAT-GILZ decreased the influx of neutrophils to the lungs and the concentrations of pro-inflammatory cytokines, resulting in reduced lung injury induced by *S. pneumoniae* infection. Of note, it has been shown that the intense inflammatory response during severe pneumococcal pneumonia, rather than enhancing clearance of bacteria, is associated with unrestrained pathogen proliferation [11]. In the present work, GILZ modulation of host responses led to control of inflammation-related bystander damage and significantly decreased bacteria counts in the airways of mice. Therefore, here we described another proresolving bioaction of GILZ–the induction of bacteria phagocytosis by macrophages.

These results are similar to findings of reduced bacterial counts in blood and increased phagocytosis in mice overexpressing GILZ when subjected to the cecal ligation and perforation (CLP) model of polymicrobial sepsis [17]. In addition, overexpression of GILZ specifically on monocytes and macrophages, enhanced bacterial clearance by phagocytosis, reduced pro-inflammatory cytokines and improved survival of mice experiencing CLP [18]. Together, the data advance the evidence for GILZ-mediated improvements in macrophage antibacterial functions during infections. Conversely, a recent study has shown that downregulation of GILZ enhanced inflammatory mediators and increased macrophage phagocytosis of *S. typhimurium* in vitro [49]. Whether this contrasting finding is related to the bacteria strain used is yet to be described.

In conclusion, we demonstrate a protective effect of endogenous and exogenous GILZ upon inflammation, bacteria proliferation, and tissue damage, associated with reduction in lethality after GILZ treatment. As increased bacterial DNA can impair neutrophil apoptosis and efferocytosis, further perpetuating inflammation during infection [50,51], reducing bacterial counts and promoting efferocytosis by TAT-GILZ induces crucial events for inflammation regulation and restoration of tissue homeostasis. Altogether, these findings suggest that harnessing GILZ might represent a beneficial adjunctive therapy, in addition to antibiotics, in the treatment of severe pneumonia.

## Data Availability

Data sets generated are available in the current manuscript.

## Supplementary Materials

The following supporting information can be downloaded at: https://www.mdpi.com/article/10.3390/cells11030532/s1, Figure S1: Intranasal challenge with LPS leads to an intense neutrophilic infiltrate into the alveoli.
