# Supplementary material for: Glucocorticoid-Induced Leucine Zipper Alleviates Lung Inflammation and Enhances Bacterial Clearance during Pneumococcal Pneumonia
Source: Cells. 2022 Feb 3;11(3):532. doi: 10.3390/cells11030532 (PMC8834062; doi:10.3390/cells11030532)
Supplement: Supplementary file 1 [file cells-11-00532-s001.zip › cells-1545318-supplementary.pdf]

cells

MDPI

Supplementary material

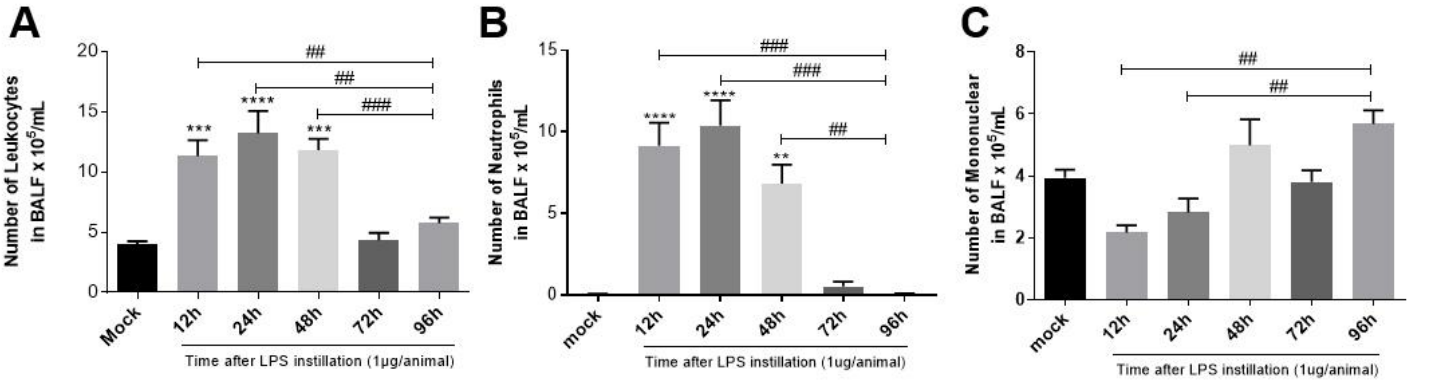

Supplementary Figure S1. Intranasal challenge with LPS leads to an intense neutrophilic infiltrate into the alveoli. C57BL/6 WT mice were stimulated with LPS (1 µg, i.n.) and euthanized 12, 24, 48, 72 and 96 h later. BAL was harvested to quantify the numbers leukocytes (**A**), neutrophils (**B**) and macrophages (C). Data are mean ± SEM of N = 4-5 animals per group. *P < 0.05 ***P < 0.001 or ****P < 0.0001 when compared to the mock group (saline instilled); or as indicated by $^{\#}P < 0.05$, $^{\#\#}P < 0.01$, $^{\#\#\#}P < 0.001$ or $^{\#\#\#\#}P < 0.0001$ when comparing LPS-challenged mice.
